# Investigation of the Underlying Mechanism of Huangqi-Dangshen for Myasthenia Gravis Treatment via Molecular Docking and Network Pharmacology

**DOI:** 10.1155/2023/5301024

**Published:** 2023-02-08

**Authors:** Miao Liu, Jing Lu, Yujuan Chen, Dongmei Zhang, Wei Huang, Mengqi Shi, Yibin Zhang, Tong Wu, Zhuming Chen, Lei Wu, Xinzhi Chen, Jian Wang

**Affiliations:** ^1^College of Chinese Medicine, Changchun University of Chinese Medicine, Changchun, Jilin, China; ^2^The First Affiliated Hospital of Changchun University of Chinese Medicine, Changchun, Jilin, China; ^3^Changchun University of Science and Technology, Changchun, Jilin, China; ^4^School of Pharmaceutical Sciences, Jilin University, Changchun, Jilin, China; ^5^The First Clinical Hospital Research Institute of Jilin Academy of Chinese Medicine, Changchun, Jilin, China

## Abstract

The herbal pairing of Huangqi and Dangshen (HD) is traditional Chinese herbal medicine and has been widely used in China, especially to treat myasthenia gravis (MG). However, the mechanism of HD on MG is unclear. *Aim of the Study*. This study aims to investigate HD's possible role in MG treatment. *Materials and Methods*. The TCMSP database was used to identify the active chemicals and their targets. The GeneCards, DisGeNET, and OMIM databases were used to search for MG-related targets. The STRING database was employed in order to identify the common PPI network targets. We next utilised Cytoscape 3.8.2 for target identification and the DAVID database for gene ontology (GO) function analysis as well as Encyclopaedia of Genomes (KEGG) pathway enrichment analysis on the selected targets. The AutoDock Vina software was used to test the affinity of essential components with the hub gene before concluding that the primary targets were corrected through molecular docking. *Results*. 41 active compounds were screened from HD, and the number of putative-identified target genes screened from HD was 112. There were 21 target genes that overlapped with the targets of MG, which were postulated to be potential treatment targets. Through further analysis, the results showed that the active compounds from HD (such as 7-methoxy-2-methylisoflavone, quercetin, luteolin, Kaempferol, and isorhamnetin) may achieve the purpose of treating MG by acting on some core targets and related pathways (such as EGFR, FOS, ESR2, MYC, ESR1, CASP3, and IL-6). Molecular docking findings demonstrated that these active molecules have a near-perfect ability to attach to the primary targets. *Conclusion*. Through network pharmacology, the findings in this study provide light on the coordinated action of several HD formula components, targets, and pathways. It provided a theoretical basis for further study of HD pharmacological action.

## 1. Introduction

Antibodies attached to components of the neuromuscular junction, such as the acetylcholine receptor (AChR), are the primary cause of myasthenia gravis (MG). More than 700,000 individuals throughout the globe are thought to be affected by the condition, which has an incidence of 0.3 to 2.8 per 100,000 [1]. MG has different rates of occurrence for different age groups, sexes, and cultures. Estimates place the median worldwide prevalence rate at 10 cases per 100,000 people. Annual incidence rates across European countries vary from 0.4% in Norway to 2.1% in Italy. Predictions put the annual rate in Australia at 1.9% per 100,000 people. The MG incidence rate in Japan is between 0.69 and 0.87 per 100,000 people, which is quite close to the MG incidence rate in Korea, which is also 0.69 per 100,000 people [2]. The clinical manifestations include drooping eyelids, weakness of the limbs, muscle wasting, and dysphagia. The symptoms worsen after exercise. Western medicine treatment mostly uses glucocorticoids, immunosuppressants, cholinesterase inhibitors, plasma exchange, thymectomy, etc [3]. Although it has a curative effect, there are certain adverse reactions to long-term application.

Traditional Chinese medicine (TCM) has been widely studied because of its stable curative effect, no drug resistance, and fewer toxic and side effects. The herbal pairing of Huangqi and Dangshen (HD) is traditional Chinese herbal medicine and has been widely used in China, especially to treat myasthenia gravis (MG). Huangqi (HQ) is the root of *Astragalus membranaceus (Fisch.) Bge. var. mongholicus (Bge.) Hsiao* and has been found to be rich in flavonoids, saponins, polysaccharides, and amino acids. Dangshen (DS) is the root of *Codonopsis pilosula (Franch.) Nannf* and is composed of several bioactive phytochemicals including lithospermic acid, protocatechuic acid, danshensu, caffeic acid, protocatechualdehyde, rosmarinic acid, salvianolic acid A-C, cryptotanshinone, dihydrotanshinone I, tanshinone IIa, and tanshinone I. However, it is not known how HD affects MG. HD is a very important supplement of TCM treasure house, both of which have the effect of invigorating the spleen and replenishing Qi. It can be used for diseases such as myasthenia gravis, heart failure, and hypotension caused by Qi deficiency. Qi deficiency syndrome is the main pathogenesis of myasthenia gravis, which runs through the whole process of myasthenia gravis. Therefore, invigorating Qi is the basic principle of treatment for MG with Qi deficiency syndrome. Clinically, HD has been widely used for the treatment of myasthenia gravis [4]. Studies have found that the main active components in HQ are saponins, flavonoids, and astragalus polysaccharides, which are rich in immune active substances. Because of their significant immune regulation, they can improve the body's immunity, thereby reducing the use of antibiotics [5, 6]. DS extract can significantly increase the number of many cells, such as white blood cell, platelet count, reticulocyte, and bone marrow nucleated cell, and increase the ratio of CD4+/CD8+, spleen coefficient, thymus coefficient, and the secretion of TNF *α*. Therefore, DS extract can enhance the hematopoietic and immune function of mice [7]. Because of its wide range of pharmacological activities, DS extract also has neuroprotection, regulation of gastrointestinal function, regulation of endocrine function, antiaging, and antioxidation properties [8]. However, the specific mechanism of action and related pathways related to HD treatment of MG remains to be further explored. The immense pharmacological outcome associated with the use of HD makes us to undertake this study to explore the underlying mechanism of Huangqi-Dangshen for myasthenia gravis treatment via molecular docking and network pharmacology.

**Figure 1:**
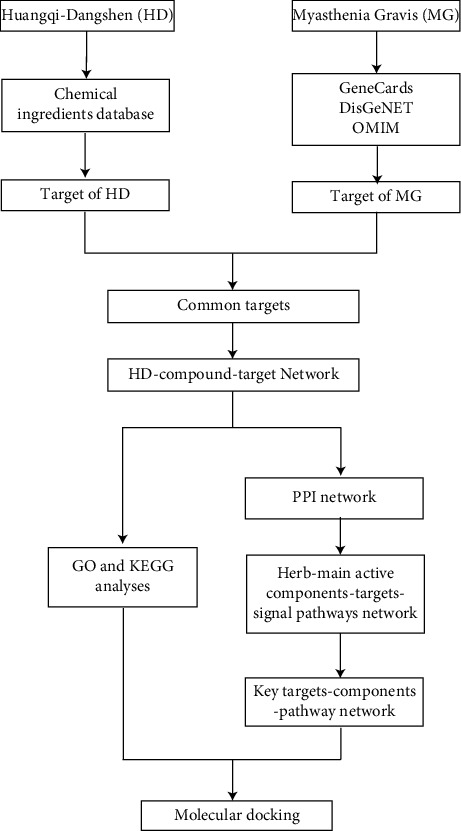
Flowchart of analysis performed in this research.

Network pharmacology is a complex system that describes the interaction of “drug (compound)-target (gene)-disease.” It analyzes the network of biological systems through bioinformatics databases, combined with systems biology, bioinformatics, pharmacological analysis, computational biology, and other multidisciplinary theories. It is a new method to analyze the target and mechanism of drug intervention in diseases from multiple perspectives [9]. When it comes to predicting the affinity and binding mechanisms of ligands and proteins, molecular docking is an invaluable tool. By predicting the binding mode and binding-free energy between receptor molecules and ligand molecules, the function and mechanism of action can be studied. It has been extensively employed in the research of TCM's particular targets and compound active elements from the recipe [10]. We employed network pharmacology and molecular docking technologies in this research to investigate the mechanism of HD therapy for MG, resulting in a novel idea for clinical implementation.  Figure 1 shows the research flow diagram.

## 2. Methods

### 2.1. Composition of TCM Compounds

Using the keywords “Astragalus” and “Codonopsis,” the TCMSP (https://tcmspw.com/tcmsp.php) [11] database was used to obtain information on the drug composition. The bioavailability (OB) was greater than 30%, and the main active ingredients were screened out if the drug-like property (DL) was greater than 0.18.

### 2.2. Screening of Target Proteins and Disease Targets of Active Ingredients of TCM

(1). Create a database of TCM active components using the TCMSP database to anticipate the target proteins of the active substances. (2). Search for myasthenia gravis (MG)-related genes in GeneCards (https://www.genecards.org/) [12], DisGeNet (https://www.disgenet.org/) [13], and the OMIM database (https://www.omim.org/) [14] using the search phrase “disease name.” The duplicated or invalid genes were eliminated from the disease target database, which was created by combining the data from the three databases.

### 2.3. Screening and Subnetwork Analysis of PPI Network and Hub Genes

The targets of each active component of TCM and the disease target were intersected. There were only a limited number of molecules that intersected in the collection; thus, the target molecules were uploaded to the STRING database (https://string-db.org) [15]. Cytoscape is an open platform with a variety of plugins to increase the visualization choices and the power of network analysis. Multiple layers of information, including protein function annotations, genome-wide studies, and large scale, can be placed on the interactome using Cytoscape, making it simple to access the network's graphical representation. Many Cytoscape plugins allow us to rank and grade the nodes according to network properties. For directed and/or undirected networks, CentiScaPe and NetworkAnalyzer, respectively, compute a number of topological network parameters. These plugins offer more centrality metrics than other regularly used tools. Different approaches concentrate on various topological properties or comparable traits with various scoring schemes. More network properties are utilised to facilitate network analysis for biologists. Import the constructed PPI network information into the Cytascape software, and the PPI network associated with the intersection molecules were screened out. Then, the cytoHubba plugin's topology algorithm is used to predict which protein nodes in the network are important and which of their subnetworks are important, with a confidence level of ≥0.700. Five parameters were used to jointly screen hub gene in this study, which was DEGREE, MNC, MCC, EPC, and CLONESS, and perform visual process. The plug-in MCODE was used for cluster analysis. Clusters of genes were discovered, subnetworks were developed, and differential genes were extracted from each cluster. The primary biological processes of the targets in each subnetwork were also analyzed.

### 2.4. KEGG Pathway Analysis, GO Classification Enrichment Analysis, and Disease Enrichment Analysis

Import the screened Hub gene into DAVID 6.8 database (https://david.ncifcrf.gov) [16], and selected “Humo space” for sepecies, then analyzed with GO analysis and KEGG pathway analysis (*P* < 0.05). Key targets' signalling pathways and important biological processes were examined. The significance of an association between the target gene set and a given gene ontology or biological pathway was determined using a hypergeometric distribution model, as shown in the following equation:

(1) $$\begin{array}{c} P=1-\overset{k-1}{\underset{i=0}{\sum }}\frac{\left(\begin{array}{c} M\\ i \end{array}\right)\left(\begin{array}{c} N-M\\ n-i \end{array}\right)}{\left(\begin{array}{c} N\\ n \end{array}\right)}\text{.} \end{array}$$

Specifically, *n* is the number of genes identified as being targets of HD, *M* is the number of genes annotated to specific GO terms or pathways, N is the total number of genes in the reference set, and *k* is the number of genes that are shared between HD-target genes and the reference set.

With the parameters set at P 0.5 and a large number of target enrichment, 247 molecules were added into the DAVID database for GO enrichment study and analyzed for GO enrichment. Key biological processes include the positive regulation of gene expression and transcription from the RNA polymerase II promoter, as well as the response to drugs, the negative regulation of the apoptotic process, cell proliferation, and so on. Finally, the findings of the disease enrichment were exported.

### 2.5. “Active Ingredient-PotentialTarget-Action Pathway” Network Construction

Cytoscape v3.8.2 was used to create the “active ingredient-potentialtarget-action route” network [17]. Active components such as quercetin, kaempferol, lignan, isorhamnetin, and 7-methoxy-2-methylisoflavone, target proteins such as EGFR, FOS, ESR1, ESR2, MYC, CASP3, IL6, and other genes, and pathways such as PI3K-Akt and PI3K/Akt/mTOR corresponding to TCMs make up the network.

### 2.6. Molecular Docking of Key Targets and Components

An AutoDock [18] programme is used to examine and dock molecular docking target molecules. The AutoDock tools are used to construct the docking grid box of the crystal structure for the target. AutoDock Vina is used to select the combination in the docking structure. Molecules with the lowest energy and the binding effect can be observed by comparison with the original ligand and intermolecular interactions. Discovery Studio is used for docking, preprocessing, and visualization. TMC's small molecule compounds' 3D structures may be obtained from PubChem (https://pubchem.ncbi.nlm.nih.gov/) using the PubChem ID. From the PDB (http://www.rcsb.org/pdb/home/home.do) database [19], the high-resolution crystal structures of important targets can be obtained.

## 3. Results

### 3.1. Screening of TCM Compound Molecules

41 kinds of main chemical components in Astragalus + Codonopsis (HD) were obtained through the TCMSP database, including 20 from HQ and 21 from DS. The corresponding information for the screened active ingredients is shown in Table 1.

### 3.2. Screening of Targets Related to Myasthenia Gravis in TCM Compounds

**Figure 2:**
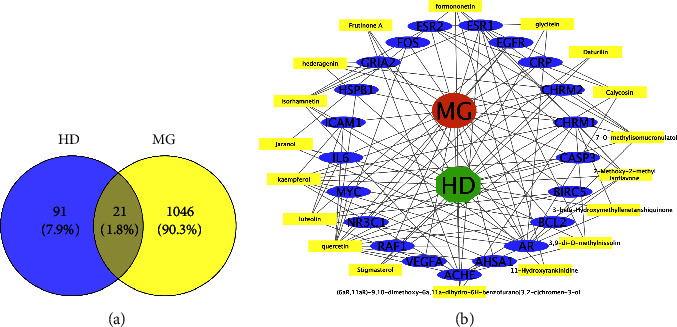
The interaction network diagram of TCM in treating diseases. (a) A Venn diagram of disease targets and drug targets; (b) a TCM component-target interaction network using myasthenia gravis and HQ-DS as the component and target, respectively.

A total of 97 HQ active ingredient targets and 51 DS active ingredient targets were identified using the target database's prediction findings. A total of 112 targets were gathered from the merged and deduplicated data sets. The GeneCards database yielded 918 disease-related targets; the DisGeNET database returned 336; the OMIM database returned seven; all based on the search keywords. After integrating and deduplicating, we had a total of 1067 targets. In order to create the Venn diagram, 112 TCM components and 1067 disease targets were mapped out (Figure 2(a)). A TCM-drugtarget-disease interaction network was established with the help of 21 crossover compounds uncovered (Figure 2(b)).

### 3.3. Construction of Protein-Protein Interaction (PPI) Network and Screening of Key Targets

**Figure 3:**
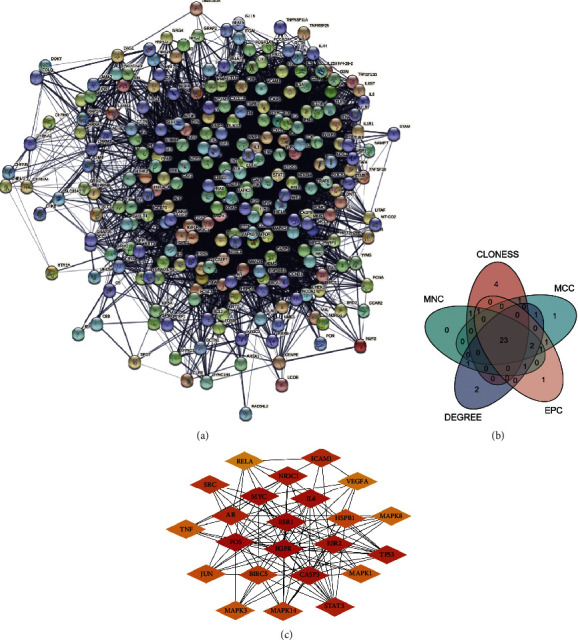
Protein interaction network diagram. The top 30 targets of the five algorithms MNC, DEGREE, MCC, CLONESS, and EPC are shown in (a) all target protein interaction networks, (b) a Venn diagram, and (c) important target protein interaction network diagrams.

There are only 21 intersection molecules between the action targets of TCM ingredients and disease targets, which are relatively small. These target compounds were then submitted to STRING to acquire information on their interactions with proteins. And a total of 21 molecules from the intersections were isolated. Then, the linked protein interaction information was obtained and formed a PPI network map (Figure 3(a)), in which there were a total of 247 molecules. Cytoscape was used to create a PPI network map based on the imported protein interaction data. Screening was based on MNC, MCC, CLONESS, DEGREE, and EPC criteria (Figure 3(b), Table 2). Select the key genes of each algorithm and intersect them with the top 30 results of each algorithm. 23 key targets were obtained (Figure 3(c), Table 3), finally. As the color of the red area is darker and the corresponding degree value is larger, there are additional potential targets that can work along with the projected disease-related targets.

### 3.4. Subnetwork Analysis

**Figure 4:**
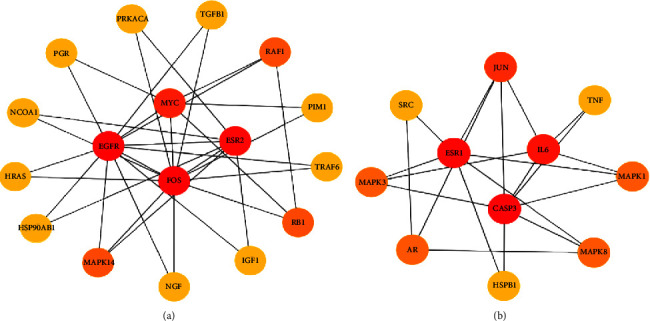
Subnetwork diagram. (a) Subnetwork diagram centered on EGFR, FOS, ESR2, and MYC. (b) Subnetwork diagram centered on ESR1, CASP3, and IL6.

MCODE subnetwork analysis is used to find more closely connected genomes in the network. The point with the highest weight obtained by weighted calculation is set as the seed. Starting from the seed, it moves outward, recursively, to find nodes that can join the subnetwork. The closer the target is to the center, the more important it is. Subnetwork 1 is centered on EGFR, FOS, ESR2, and MYC, and its important targets, such as MAPK14 and RAF1, are targets related to the MAPK pathway, indicating that subnetwork 1 is closely related to it (Figure 4(a)). The core of subnetwork 2 is ESR1, CASP3, and IL6, which are closely related to the TNF signalling pathway and the hepatitis B pathway (Figure 4(b)).

### 3.5. GO Classification and Enrichment Analysis Results

**Figure 5:**
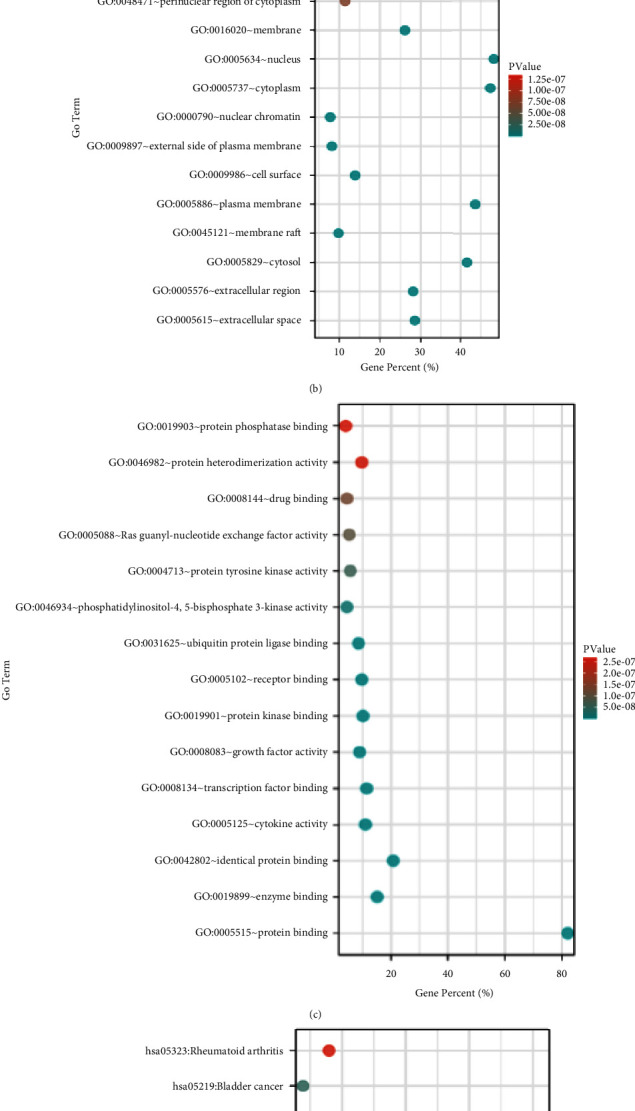
GO-KEGG enrichment results (Top15). (a) BP analysis; (b) MF analysis; (c) CC analysis; (d) KEGG analysis.

A total of 247 molecules were entered into the DAVID database for GO enrichment analysis and evaluated for GO enrichment under the parameters of *P* < 0.5 and a large number of target enrichments, as shown in Figures 5(a)–5(c), Table 4. In the biological process, the key targets are concentrated in the positive regulation of gene expression, and transcription from RNA polymerase II promoter, response to drug, negative regulation of the apoptotic process, and cell proliferation, and positive regulation of cell proliferation, etc. Among the cell components, the most targets are in the cytoplasm, followed by the plasma membrane, cytosol, extracellular space, the nucleus, etc. Molecular functions mainly involve protein, enzyme binding, cytokine activity, transcription factor binding, etc.

### 3.6. KEGG Pathway Analysis

Potential targets were subjected to KEGG pathway enrichment analysis (*P* < 0.05) through the DAVID 6.8 data platform, as shown in Figure 5(d) and Table 5. Hepatitis B, the PI3K-Akt signalling pathway, cancer-related and proteoglycan cancer pathways, inflammatory bowel disease, Chagas disease, prostate cancer, osteoclast differentiation, colorectal cancer, and the TNF signalling pathway are the top ten pathways.

### 3.7. Results of Network Construction of “Active Ingredients-KeyTargets-Action Pathways” of TCM

**Figure 6:**
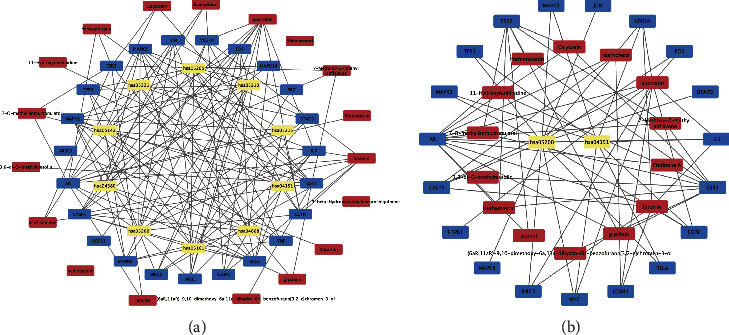
Medicinal material-component-target-pathway diagram. (a) component-target-pathway diagram; (b) key target-component diagram (red is the component, blue is the target, and yellow is the pathway).

As a starting point, the herbs in the recipe were loaded into Cytoscape 3.8.2 software, together with possible targets, active components, and signalling pathways evaluated in the compound (Figure 6(a)). Further calculations were performed using cytoHubba's MCC algorithm to obtain the closest association of components to key targets (Figure 6(b)).

### 3.8. Molecular Docking to Simulate the Interaction between the Target and Related Compounds

**Figure 7:**
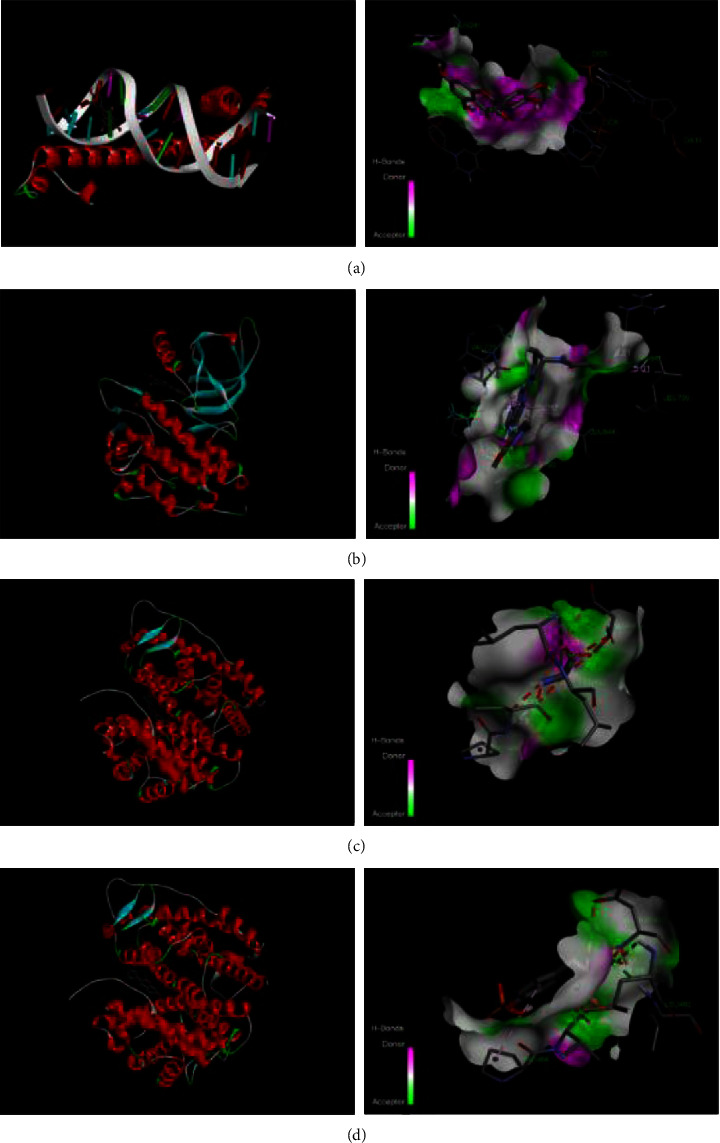
Molecular docking simulation diagram of target and compound. (a) quercetin-FOS; (b) luteolin-EGFR; (c) isorhamnetin-ESR2; (d) 7-methoxy-2-methylisoflavone-ESR2.

The five compounds with the most key target genes were docked with their key target genes, and it was found that their docking binding energies with the target were all <−5, indicating that the two had a better binding effect. Among them, quercetin and FOS, luteolin and EGFR, isorhamnetin, and ESR2, the lowest binding energies are possessed by 7-methoxy-2-methylisoflavone and ESR2, and the binding mechanism is shown in Figure 7 and Table 6.

## 4. Discussion

In this study, 41 major chemical components and 21 targets related to myasthenia gravis from HQ and DS for HD were selected. Quercetin, kaempferol, lignan, isorhamnetin, and 7-methoxy-2-methylisoflavone were the most important active components in the treatment of MG through active component screening and complex target network analysis. Studies have shown that quercetin, kaempferol, lignan, isorhamnetin, and 7-methoxy-2-methylisoflavone have active effects, such as anti-inflammatory, immunomodulatory, antioxidative stress, and neuroprotective [20–23]. The JAK-STAT pathway may be blocked by quercetin, which inhibits the release of IL-12 by T cells, as well as the proliferation of activated T cells and the development of Th1 cells [24]. Kaempferol can inhibit the release of inflammatory factors by inhibiting MAPK pathways activated by extracellular signal-regulated kinases 1 and 2 (Erk1/2) [25]. It can also effectively interfere with the reverse transcription of STAT3 and inhibits the activation of inflammatory factors by blocking the Tyk-STAT signalling pathway [26]. Lignocaine can significantly reduce the levels of IL-6 and TNF-*α* cytokines in serum and act as an anti-inflammatory and neuroprotective agent [27]. Isorhamnetin pretreatment can inhibit caspase-3 activation and significantly increase AKT serine/threonine kinase 1 (AKT) and phosphorylation of phosphatidylinositol 3-kinase (PI3K) in cells [28]. 7-methoxy-2-methylisoflavone belongs to the group of flavonoids. And flavonoids can reduce the expression level of inflammatory mediators. In addition, flavonoids can also modulate the imbalance of Th1/Th2 cytokines, thereby playing a role in regulating immunity [29]. An unbalanced ratio of Th1 to Th2 cytokines is thought to have a role in immune-mediated illness genesis and progression [30]. In summary, HD may treat myasthenia gravis through anti-inflammatory, immunomodulatory, and neuroprotective effects and may inhibit inflammatory cytokines through a variety of inflammatory signalling pathways.

In order to predict the mechanism of action of HD for MG, the intersecting targets were screened in the PPI network by cytoHubba and MCODE plugins, and the 7 core targets that were obtained were EGFR, FOS, ESR1, ESR2, MYC, CASP3, and IL-6. It was found that HD could influence the occurrence and progression of MG through biological processes such as the MAPK cascade, the extrinsic apoptotic signalling pathway in the absence of ligand, immune response, inflammation response, and negative regulation of apoptotic processes, as shown by GO enrichment analysis. Recent studies have reported that EGFR can be expressed in thymoma, which is an important cause of myasthenia gravis, and that EGFR overactivates the downstream PI3K/Akt/mTOR signalling pathway, inhibits apoptosis, and stimulates tumor growth [31, 32]. EGFR can also activate the MAPK signalling cascade, which in turn stimulates transcription factors that drive the expression of genes associated with tumor invasion and metastasis [33]. Fosl1 and Fosl2 are members of the Fos family. INKT cells were shown to grow when Fosl2 was targeted specifically to CD4+ T lymphocytes. And Fosl2 regulates the normal development and cellular function of iNKT cells and is involved in the process of iNKT cell selection [34] and significantly decreased after treatment [35]. In autoimmune myasthenia gravis, which is more frequent in women than in men, the ESR1 gene expressing estrogen receptor *α* (ER*α*) and the ESR2 gene expressing estrogen receptor *β* (ER*β*) can mediate multiple physiological effects of estrogen [36]. A recent study carried out by Chao et al. has reported that EGFR and MYC are key targets in MG on performing molecular docking and systems pharmacology analysis considering nux vomica [37]. Immune cells include B cells, CD4+ T cells, CD8+ T cells, NK cells, and plasma cell-like DCs, which have been shown to express high levels of the ESR1 and ESR2 genes. And estrogen receptors regulate autoimmune responses through positive or negative regulation of proinflammatory cytokines [38, 39]. Cystathione-3 (CASP3) is a key executive enzyme of apoptotic function, and research has shown that CASP3 expression increases with the progression of MG staging, and apoptosis in the thymus of MG patients is closely associated with CASP3 activation [40, 41]. Lack of CASP3 prevents skeletal muscle atrophy by inhibiting apoptotic signalling in denervated muscles [42]. Plasmacytoid dendritic cells (pDC) mainly produce IFN-1. When cells are induced by viral infection or tumor cells, the body produces endogenous IFN-1, which enhances innate and acquired immunity, stimulates the activity of cytotoxic T cells and NK cells, and promotes antibody production. Downregulation of Myc in pDC cell lines can lead to a significant increase in the secretion of IFN-1 which in turn triggers inflammatory and adaptive autoimmune responses [43, 44]. The proinflammatory cytokine interleukin-6 (IL-6) has a wide range of applications. As Tfh, Th17, and regulatory T cells' abilities are suppressed by IL-6, the development of autoimmune disorders is facilitated (Treg) [45]. Studies have shown that anti-IL-6 antibody treatment can reduce the level of anti-AChR antibody in EAMG model rats. It can inhibit Th1, Th17, and B cell responses and reduce the severity of MG as well. Muscle atrophy and elevated levels of histone proteases were the results of transgenic mice overexpressing IL-6. Immunity and muscle proteolysis seem to be regulated by IL-6, according to these studies [46, 47]. Clinical studies have shown that the level of IL-6 in serum is significantly elevated in MG patients positive for anti-AChR antibodies [48, 49]. In addition, tocilizumab monoclonal antibody blocks the binding of IL-6 to the IL-6R receptor, thereby inhibiting classical and trans-signalling and proinflammatory factor activity. It is expected to be an alternative therapeutic option in cases where rituximab monoclonal antibodies are ineffective [50]. The results showed by Bahauddin et al. revealed that histone deacetylases (HDACs) had different effects on inflammation depending on the isoform, and they also uncovered a large number of genes involved in several inflammatory like IL-6 and IL-21 and autoantibody pathways like acetylcholine receptor (AChR)-specific autoantibodies, in EAMG that are controlled by HDACs [51]. So the above-mentioned intersecting targets EGFR, FOS, ESR1, ESR2, CASP3, MYC, and IL-6 are key targets of action in the treatment of MG with HD.

HD may serve a therapeutic function in MG via the PI3K-Akt signalling pathway, inflammatory bowel illness, according to the findings of the KEGG enrichment study. Cell proliferation, differentiation, metabolism, and death are all regulated by the PI3K-Akt signalling system, according to research. Different hormones, growth factors, and cell-to-cell contacts frequently trigger this regulation [52]. It is believed that the loss of Treg function and Th17 differentiation and activation are responsible for the onset of MG in humans. It is possible to suppress the development of Treg cells by activating PI3K and mTORC1 in CD4+ T cells while promoting the differentiation and expression of Th17 cells [53, 54]. As a downstream target of mTORC1, HIF-1*α* enhances Th17 expression and regulates Th17 differentiation by increasing aerobic glycolytic activity required for rapid T-cell expansion [55, 56]. Myasthenia gravis patients' immune systems may be influenced by the PI3K/Akt/mTOR signalling pathway, according to these studies. Inflammatory bowel illness has been linked to signal transducer and activator of transcription 3 (STAT3), according to research. When STAT3 or STAT3 phosphorylation expression is increased in human IBD, STAT3 is activated in an IL-6dose-dependent relationship. Phosphorylated STAT3 can upregulate transcription factors in EAMG model rats' Tfh cells and improve humoral immune response [57, 58]. A recent study carried out by Li et al. has reported that regulators of the PI3K/AKT/mTORC1-HIF-1*α* have a significant role in managing MG patients and concluded that the mTOR-HIF-1*α* signalling could be the possible immune metabolism reprogramming checkpoint of MG [59].

After docking the five major components of HD with their key target genes, it was found that their docking binding energy with the target was all <−5. It indicated that the five key components showed a strong affinity with the key target genes. Therefore, HD is expected to be a major component of novel natural drugs for the treatment of MG.

## 5. Conclusions

In summary, the results of the network pharmacological analysis indicate that HD mainly acts on EGFR, FOS, ESR1, ESR2, MYC, CASP3, IL-6, and other genes through various active components, such as quercetin, kaempferol, lignan, isorhamnetin, and 7-methoxy-2-methylisoflavone. These components treat myasthenia gravis through anti-inflammatory, immunomodulatory, and inhibiting muscle cell apoptosis. Molecular docking verified the treatment effect of HD on MG. The mechanism of action of HD in the treatment of myasthenia gravis is through the combined action of various major active ingredients from the formula with potential core targets. This study laid the foundation for the in-depth study of the pharmacological mechanism of HD in the treatment of myasthenia gravis.

## 6. Limitations and Recommendations

Our study results showed significant efficacy of HD against MG by interacting and altering several gene expressions. Besides these wonderful results, our study lacks practical experimentation. To prove these facts as an outcome of network pharmacological analysis, experimentation is recommended and could be a step forward.

## Figures and Tables

**Table 1:** Chemical composition of TCM.

Mol ID	Molecule name	OB%	DL	Source
MOL000211	Mairin	55.38	0.78	Huangqi
MOL000239	Jaranol	50.83	0.29	Huangqi
MOL000296	Hederagenin	36.91	0.75	Huangqi
MOL000033	(3S,8S,9S,10R,13R,14S,17R)-10,13-dimethyl-17-[(2R,5S)-5-propan-2-yloctan-2-yl]-2,3,4,7,8,9,11,12,14,15,16,17-dodecahydro-1H-cyclopenta[a]phenanthren-3-ol	36.23	0.78	Huangqi
MOL000354	Isorhamnetin	49.60	0.31	Huangqi
MOL000371	3,9-di-O-methylnissolin	53.74	0.48	Huangqi
MOL000374	5′-hydroxyiso-muronulatol-2′,5′-di-O-glucoside	41.72	0.69	Huangqi
MOL000378	7-O-methylisomucronulatol	74.69	0.30	Huangqi
MOL000379	9,10-dimethoxypterocarpan-3-O-*β*-D-glucoside	36.74	0.92	Huangqi
MOL000380	(6aR,11aR)-9,10-dimethoxy-6a,11a-dihydro-6H-benzofurano[3,2-c]chromen-3-ol	64.26	0.42	Huangqi
MOL000387	Bifendate	31.10	0.67	Huangqi
MOL000392	Formononetin	69.67	0.21	Huangqi
MOL000398	Isoflavanone	109.99	0.30	Huangqi
MOL000417	Calycosin	47.75	0.24	Huangqi
MOL000422	Kaempferol	41.88	0.24	Huangqi
MOL000433	FA	68.96	0.71	Huangqi
MOL000438	(3R)-3-(2-hydroxy-3,4-dimethoxyphenyl)chroman-7-ol	67.67	0.26	Huangqi
MOL000439	Isomucronulatol-7,2′-di-O-glucosiole	49.28	0.62	Huangqi
MOL000442	1,7-Dihydroxy-3,9-dimethoxy pterocarpene	39.05	0.48	Huangqi
MOL000098	Quercetin	46.43	0.28	Huangqi
MOL000006	Luteolin	36.16	0.25	dangshen
MOL000449	Stigmasterol	43.83	0.76	dangshen
MOL001006	Poriferasta-7,22E-dien-3beta-ol	42.98	0.76	dangshen
MOL002140	Perlolyrine	65.95	0.27	dangshen
MOL002879	Diop	43.59	0.39	dangshen
MOL003036	ZINC03978781	43.83	0.76	dangshen
MOL003896	7-methoxy-2-methyl isoflavone	42.56	0.20	dangshen
MOL004355	Spinasterol	42.98	0.76	dangshen
MOL004492	Chrysanthemaxanthin	38.72	0.58	dangshen
MOL005321	Frutinone A	65.90	0.34	dangshen
MOL006554	Taraxerol	38.40	0.77	dangshen
MOL006774	Stigmast-7-enol	37.42	0.75	dangshen
MOL007059	3-beta-Hydroxymethyllenetanshiquinone	32.16	0.41	dangshen
MOL007514	Methyl icosa-11,14-dienoate	39.67	0.23	dangshen
MOL008391	5alpha-Stigmastan-3,6-dione	33.12	0.79	dangshen
MOL008393	7-(beta-Xylosyl)cephalomannine qt	38.33	0.29	dangshen
MOL008397	Daturilin	50.37	0.77	dangshen
MOL008400	Glycitein	50.48	0.24	dangshen
MOL008406	Spinoside A	39.97	0.4	dangshen
MOL008407	(8S,9S,10R,13R,14S,17R)-17-[(E,2R,5S)-5-ethyl-6-methylhept-3-en-2-yl]-10,13-dimethyl-1,2,4,7,8,9,11,12,14,15,16,17-dodecahydrocyclopenta[a]phenanthren-3-one	45.40	0.76	dangshen
MOL008411	11-Hydroxyrankinidine	40.00	0.66	dangshen

**Table 2:** Ranking of calculation results of cytoHubba five algorithms.

Rank	MCC	Closeness	MNC	Degree	EPC
1	EGFR	EGFR	EGFR	EGFR	EGFR
2	FOS	CASP3	CASP3	CASP3	ESR1
3	ESR1	IL6	ESR1	IL6	CASP3
4	MYC	FOS	FOS	FOS	FOS
5	IL6	ESR1	IL6	ESR1	IL6
6	CASP3	AR	AR	AR	AR
7	ESR2	MYC	MYC	ICAM1	MYC
8	TP53	JUN	ICAM1	MYC	STAT3
9	NR3C1	STAT3	ESR2	CRP	TP53
10	STAT3	TP53	NR3C1	CHRM1	ESR2
11	AR	MAPK14	CRP	ESR2	NR3C1
12	ICAM1	TNF	BIRC5	BIRC5	ICAM1
13	SRC	NR3C1	STAT3	NR3C1	JUN
14	CRP	MAPK1	TP53	BCL2	BIRC5
15	BIRC5	MAPK3	BCL2	STAT3	MAPK14
16	MAPK14	ESR2	TNF	TP53	TNF
17	HSPB1	SRC	RELA	ACHE	RELA
18	JUN	VEGFA	MAPK14	HSPB1	CRP
19	MAPK1	INS	SRC	GRIA2	SRC
20	MAPK3	MAPK8	JUN	SRC	MAPK1
21	TNF	HIF1A	MAPK1	JUN	HSPB1
22	MAPK8	NGF	VEGFA	TNF	MAPK3
23	VEGFA	MMP9	HSPB1	RELA	BCL2
24	BCL2	HSPB1	MAPK3	VEGFA	MAPK8
25	RELA	RELA	MAPK8	MAPK14	VEGFA
26	CHRM1	PTEN	RAF1	MAPK1	HIF1A
27	PTEN	ICAM1	RB1	RAF1	RB1
28	SMAD3	PRKCA	HIF1A	MAPK3	IGF1
29	SMAD4	PTGS2	INS	MAPK8	SMAD4
30	RUNX2	BIRC5	SMAD3	RB1	SMAD3

**Table 3:** Topological parameter analysis of key targets.

Name	Closeness	Betweenness	Degree
EGFR	155.91667	21055.22881	87
CASP3	138.91667	13581.81055	61
IL6	136.0	11100.0022	53
FOS	132.66667	9254.88492	50
ESR1	127.58333	5203.38585	47
AR	123.5	6788.10848	45
MYC	121.5	2737.21043	26
ICAM1	104.66667	1694.87097	26
ESR2	110.83333	866.28027	19
BIRC5	104.35	2468.86776	18
NR3C1	111.35	1192.98489	17
TP53	116.85	795.40972	12
STAT3	117.18333	1038.66281	12
HSPB1	106.68333	1341.56512	10
SRC	110.66667	3322.67413	8
MAPK14	113.01667	266.80844	8
JUN	117.58333	5100.94748	8
TNF	112.35	704.28349	8
VEGFA	110.01667	982.86802	8
RELA	105.51667	314.52161	8
MAPK1	111.01667	249.58642	7
MAPK3	111.01667	249.58642	7
MAPK8	108.35	247.61036	7

**Table 4:** GO analysis table.

Class	GO term	Description	Count	*P*-value
Biological process	GO:0045944	Positive regulation of transcription from RNA polymerase II promoter	69	1.15*E* − 28
	GO:0010628	Positive regulation of gene expression	38	3.96*E* − 26
	GO:0043066	Negative regulation of apoptotic process	44	3.81*E* − 23
	GO:0042493	Response to drug	35	9.83*E* − 21
	GO:0008284	Positive regulation of cell proliferation	41	4.87*E* − 20
	GO:0006954	Inflammatory response	34	7.69*E* − 17
	GO:0007165	Signal transduction	58	8.64*E* − 17
	GO:0045893	Positive regulation of transcription, DNA-templated	37	3.30*E* − 15
	GO:0001666	Response to hypoxia	23	5.90*E* − 15
	GO:0031663	Lipopolysaccharide-mediated signalling pathway	13	1.07*E* − 14

Cellular component	GO:0005615	Extracellular space	70	2.54*E* − 23
	GO:0005576	Extracellular region	69	2.03*E* − 18
	GO:0005829	Cytosol	102	9.14*E* − 18
	GO:0045121	Membrane raft	24	5.50*E* − 15
	GO:0005886	Plasma membrane	107	2.15*E* − 13
	GO:0009986	Cell surface	34	3.03*E* − 13
	GO:0009897	External side of plasma membrane	20	7.36*E* − 11
	GO:0000790	Nuclear chromatin	19	1.13*E* − 10
	GO:0005737	Cytoplasm	116	4.41*E* − 10
	GO:0005634	Nucleus	70	9.30*E* − 10

Molecular function	GO:0005515	Protein binding	201	8.17*E* − 24
	GO:0019899	Enzyme binding	37	1.74*E* − 21
	GO:0042802	Identical protein binding	51	2.78*E* − 20
	GO:0005125	Cytokine activity	27	3.63*E* − 19
	GO:0008134	Transcription factor binding	28	6.84*E* − 15
	GO:0008083	Growth factor activity	22	1.71*E* − 14
	GO:0019901	Protein kinase binding	25	1.03*E* − 09
	GO:0005102	Receptor binding	24	1.59*E* − 09
	GO:0031625	Ubiquitin protein ligase binding	21	6.19*E* − 09
	GO:0046934	Phosphatidylinositol-4,5-bisphosphate 3-kinase activity	11	1.70*E* − 08

**Table 5:** KEGG pathway enrichment information.

Term	Pathway	Count	*P*-value
hsa05200	Pathways in cancer	63	7.08*E* − 29
hsa05161	Hepatitis B	41	3.98*E* − 28
hsa05205	Proteoglycans in cancer	40	2.03*E* − 21
hsa04151	PI3K-Akt signalling pathway	47	3.49*E* − 18
hsa05321	Inflammatory bowel disease (IBD)	23	4.21*E* − 18
hsa05142	Chagas disease (American trypanosomiasis)	27	2.51*E* − 17
hsa05215	Prostate cancer	25	5.12*E* − 17
hsa04380	Osteoclast differentiation	29	1.17*E* − 16
hsa05210	Colorectal cancer	21	6.18*E* − 16
hsa04668	TNF signalling pathway	26	7.14*E* − 16

**Table 6:** Molecular docking results.

Compound	Target	PDB ID	Binding energy (kcal/mol)
Quercetin	EGFR	5ug9	−6.8
	CASP3	2dko	−6.6
	BIRC5	2qfa	−6.8
	AR	1t7r	−7.2
	VEGFA	3v2a	−6.3
	IL6	1alu	−6.4
	FOS	2wt7	−8.0
	MYC	6g6k	−6.3

Kaempferol	AR	1t7r	−7.6
	CASP3	2dko	−6.8

Luteolin	CASP3	2dko	−6.9
	BIRC5	2qfa	−7.3
	VEGFA	3v2a	−6.8
	AR	1t7r	−7.5
	EGFR	5ug9	−8.4

Isorhamnetin	ESR1	7baa	−7.2
	ESR2	3oll	−8.0

7-methoxy-2-methylisoflavone	ESR1	7baa	−7.1
	ESR2	3oll	−8.1

## Data Availability

The data used to support the findings of this study are included within the article.

## Abbreviations

MG: Myasthenia gravis
HD: Huangqi and Dangshen
AChR: Acetylcholine receptor
TCM: Traditional Chinese medicine
OMIM: Online mendelian inheritance in man
PPI: Protein-protein interaction
TCMSP: Traditional Chinese medicine systems pharmacology database and analysis platform
TTD: Therapeutic target database
PDB: Protein data bank.
